# Development of seismic resilience index methodology for RC structures via functionality curve

**DOI:** 10.1016/j.mex.2022.101865

**Published:** 2022-09-22

**Authors:** H'ng Chee Yin, Moustafa Moufid Kassem, Fadzli Mohamed Nazri, Ahmad Mohamad El Maissi

**Affiliations:** a School of Civil Engineering, Engineering Campus, Universiti Sains Malaysia, Penang 14300, Malaysia; b Academic Fellow, School of Civil Engineering, Engineering Campus, Universiti Sains Malaysia, Penang 14300, Malaysia

**Keywords:** Seismic resilience index, Functionality curve, Robustness, Vulnerability assessment, Time of recovery, Loss of resilience

## Abstract

Seismic resilience index (SRI) approach describes the post-seismic recovery phase of a single building by incorporating the time dimension. In recent decades, numerous quantitative frameworks have been developed to quantify earthquake resilience. The seismic resilience of existing reinforced concrete (RC) structures in Malaysia under far-field (FF) and near-field (NF) earthquake scenarios is examined in this research methodology using a seismic resilience index (SRI) and functionality curve. SRI is implemented to a four-story RC school building and a five-story RC hospital structure damaged by the 2015 Ranau earthquake. In addition, a total of 25 existing RC structures have been selected as target case studies for evaluating the SRI and presenting the results graphically using the Geographical Information System (GIS) platform. These buildings are primarily divided into three clusters: low, mid, and high rise. Non-linear dynamic analysis (NL-DA) is utilized in this approach to analyze the case studies and the selected buildings using the Finite Element platform (FE). Moreover, a group of seismic records encompassing of far-field and near-field are selected for simulation process using non-linear time history analysis (NL-THA). Eventually, Incremental Dynamic Analysis, fragility and vulnerability assessment, functionality assessment, time of recovery (T_REC_), loss of resilience (LOR), robustness (R) and SRI indicator are all part of this approach.

• SRI helps decision-makers create or suggest repair or retrofitting solutions with acceptable recovery time and cost.
• The consequences of failures for people's lives, properties, as well as the economy, will be mitigated.
• Improve the sustainability and serviceability of structures to ensure seismic safety and meet Sustainable Development Goal 11.

## Graphical abstract

**Figure:**
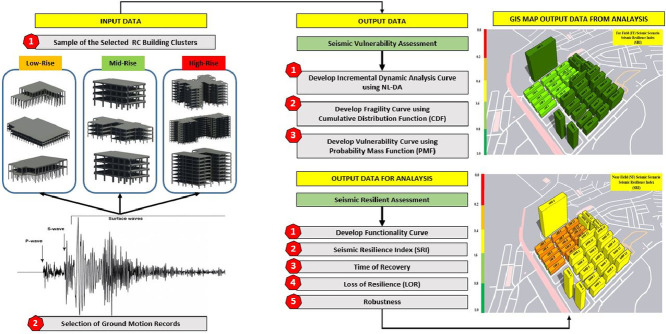

**Specifications table**

**Table:** 

Subject area:	Engineering
More specific subject area:	*Structural and Earthquake Engineering*
Name of your method:	*Seismic Resilience Index Approach*
Name and reference of original method:	M. Bruneau, S. E. Chang, R. T. Eguchi, G. C. Lee, T. D. O'Rourke, A. M. Reinhorn, M. Shinozuka, K. Tierney, W. A. Wallace, D. von Winterfeldt (2003). “A framework to quantitatively assess and enhance the seismic resilience of communities”. Earthquake Spectra 19(4): 733-752
Resource availability:	*Not Applicable*

## Method details

### Background

In seismology and earthquake engineering, resilience is an approach that introduces the time dimension to explain the post-seismic event recovery phase which covers the scopes beyond the single building to communities including both essential and non-essential systems [1]. Generally, seismic resilience is defined as the ability of a structure or a system to continue operating as normal or probably extends its serviceability after the initial damage has been repaired [2]. The concept of seismic resilience plays a critical role in the phases of rescue and recovery after seismic events by assessing the post-event functionality of the affected systems or structures. As described by Ahern [3], the concept of resilience changes the structural design requirements from ‘fail-safe’ to ‘safe-to-fail’, for further explanation, a system or a structure will not fail at a prescribed disturbance level or the failure of the system or structure at a higher disturbance level will be acceptable and allowable for a recovery phase at an optimal combination of repair time and repair cost.

**Fig 1:**
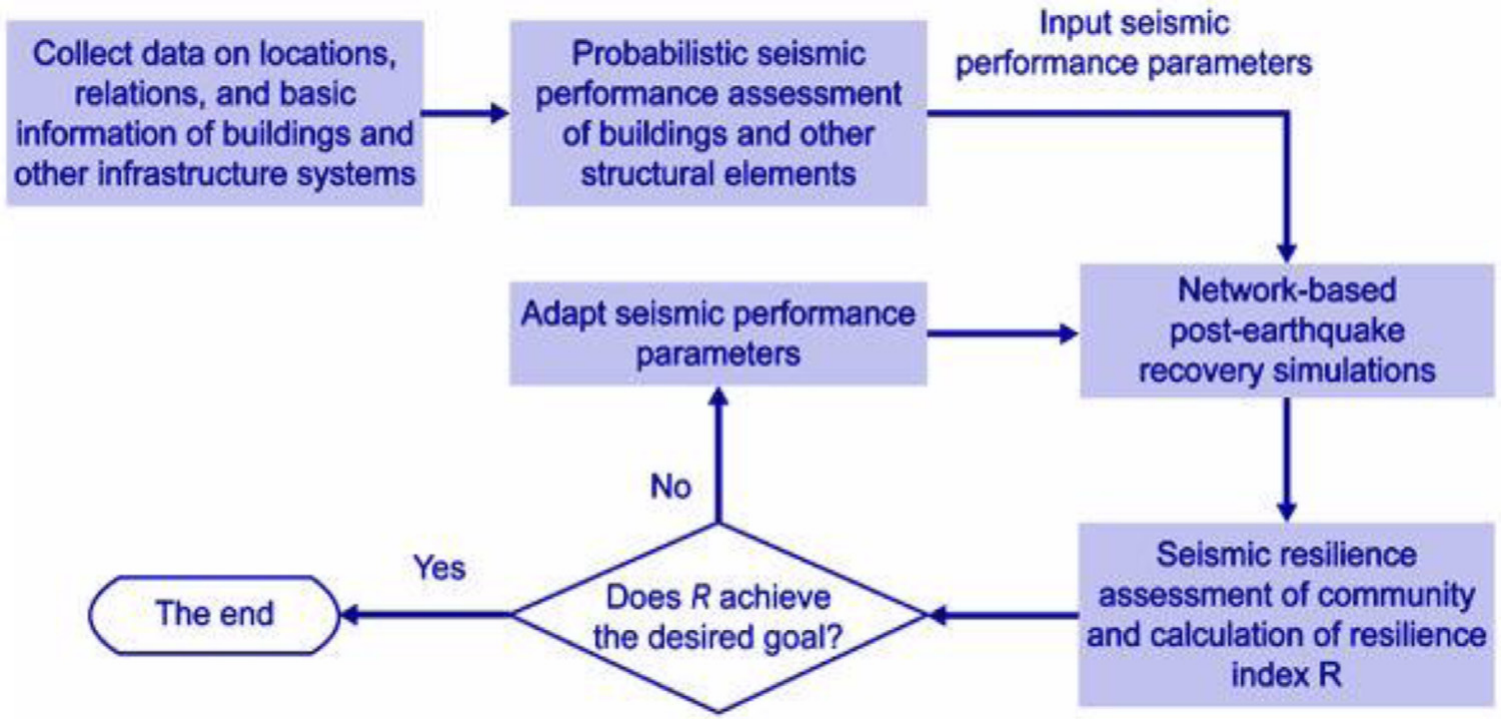
The flowchart of the framework [4].

In the early decades, community resilience was developed and evaluated qualitatively and conceptually, which was less substantial and efficient. It is possible to use the community resilience index as a starting point for tracking changes in several areas of the community through time, including socio-demographics, economy, the environment, organizational structures, and culture. Due to limited political and planning actions, the idea of community resiliency is rarely used as a tool in earthquake risk management and mitigation. This results in a defective, ineffective reconstruction procedure because of a lack of consistent, and adequate mitigating solutions. There have been various classic frameworks established recently to analyze the resiliency of communities, effective and beneficial for both single systems and the entire community. For instance, You et al. [4], established a framework that has to link between the seismic performance of structures, and the resiliency of communities. Figure depicts the framework Fig. 1.

Indeed, over the last few years, a great number of quantitative frameworks have been developed in order to evaluate the structural resilience against seismic tremors for structures that already exist in a variety of countries. Cimellaro et al. [5] offered multiple frameworks to characterize resilience quantitatively relying on an analytical approach that can be used for technological and organizational considerations. Cimellaro et al. introduced a comprehensive model to quantify the seismic resiliency of a hospital system that incorporates both loss estimation and recovery models that can be employed for critical facilities. For essential facilities, Cimellaro et al. [6] developed a comprehensive model for quantifying seismic resiliency that includes both loss estimation and restoration models. Hassan et al. [7] developed a framework for a hospital with 6- storeys that would be used to evaluate the post-earthquake functionality of the hospital system, by comprising both quantity (space availability, personnel availability, and supplies availability) and quality (satisfaction with medical treatments) portions. Nevertheless, a methodology is developed by Shang et al. [8] that contains four phases which is used to estimate the seismic resilience of hospital systems, including a seismic risk analysis, seismic hazard analysis, fragility analysis, and the determination of seismic resilience. In addition, Yarveisy et al. [9] developed straightforward resilience evaluation criteria for the purpose of quantifying and evaluating resilience based on the concepts of reliability and serviceability.

It is worth noting that, a building's resilience can be defined as its ability to withstand external threats while also regaining its functionality following damage or destruction. Structural resilience has been explored by Bruneau et al. [10] by identifying four major traits. The 4R attributes stand for resiliency, rapidity, redundancy, and resourcefulness. As stated by Lu et al. [11], there are two main known approaches to evaluate a building's structural resilience: risk and resistance analysis of structures; and rating of structural vulnerability. Risk and resistance analysis of buildings is the first method that has been developed. It is common practice to base a structure's design and information on hazards such risk and design loadings on the environment, structural element types, building materials, and GIS data for the structure. Consequently, the building's structural resilience can be estimated using the design in 1 formation. The United States Green Building Council (USGBC) recommends the Leadership in Energy and Environmental Design (LEED) analysis and planning for resilience. This process necessitates a hazard assessment of the construction project. The Building Resilience Rating Tool (BRRT) was also developed by the Insurance Council of Australia (ICA), and it was based on this methodology [12]. Moreover, BRRT rates building resilience by recognizing probable dangers. As a result, the building's materials and structural types are analyzed to determine its level of risk. On the other hand, the grading of structural resilience under a specified hazard scenario is the second method now in use. The structure's resistance can be graded or given stars based on indicators related to the 4R and other attributes that are discovered when a threat is detected. Grading systems for buildings have been proposed by the Resilience-Based Earthquake Design Initiative (REDi) as well as by the US Resiliency Council (USRC). These rating systems are based on the buildings' ability to withstand earthquakes.

The evaluation of the seismic resilience index of a structure is an essential initial step in the process of formulating a plan for repairs or upgrades. Using the functionality curves, it is possible to effectively suggest or implement a repair plan or retrofitting approach based on the results of the resilience assessment and the amount of time it takes for the system to recover. For instance, Samadian et al. [13] carried out research in Iran with the purpose of determining the seismic resilience of both newly constructed and previously used RC school buildings. The research was examined using vulnerability and fragility curves. The fundamental purpose of this research is to employ a new method that concentrates on the economic situations of the provinces through the utilization of vulnerability analysis in order to assess the losses that were caused by earthquakes. According to the findings, the SRI had a functional decline as the magnitude of the threat levels was raised higher. Titi and Biondini [14] conducted an investigation into the dependability of concrete frame structural systems that were negatively damaged by corrosion. The findings demonstrated that the constructed structures that were intended to have the same degree of functioning could display differing levels of seismic resistance over a predetermined amount of time as a direct result of the environmental variables in their immediate surroundings. Other investigations were carried out with the purpose of determining the levels of resilience and SRI possessed by various types of buildings, for example, Banerjee and Chandrasekaran [15] explored the seismic resilience of bridges when they were subjected to the influence of various hazards, whereas Alipour and Shafei [16] assessed the seismic resilience of transportation networks.

In this paper, a research methodology is applied for evaluating the seismic resiliency of damaged reinforced concrete (RC) buildings in Malaysia under Ranau ground motion at seismic intensity measure (VIII) in which a soft story failure mechanism phenomenon is appeared. An RC school building with four floors and an RC hospital building with five stories were chosen for this research so that the suggested seismic resilience index (SRI) methodology could be applied. Nevertheless, neither of these models were built to withstand the effects of seismic loading. Moreover, there are three different clusters of existing RC structures in the study area: low-, mid- and high-rise. These three building types were chosen for the case studies in order to assess the SRI and present its findings graphically using the Geographical Information System (GIS).

### Strategy adopted to develop seismic resilience index

**Fig 2:**
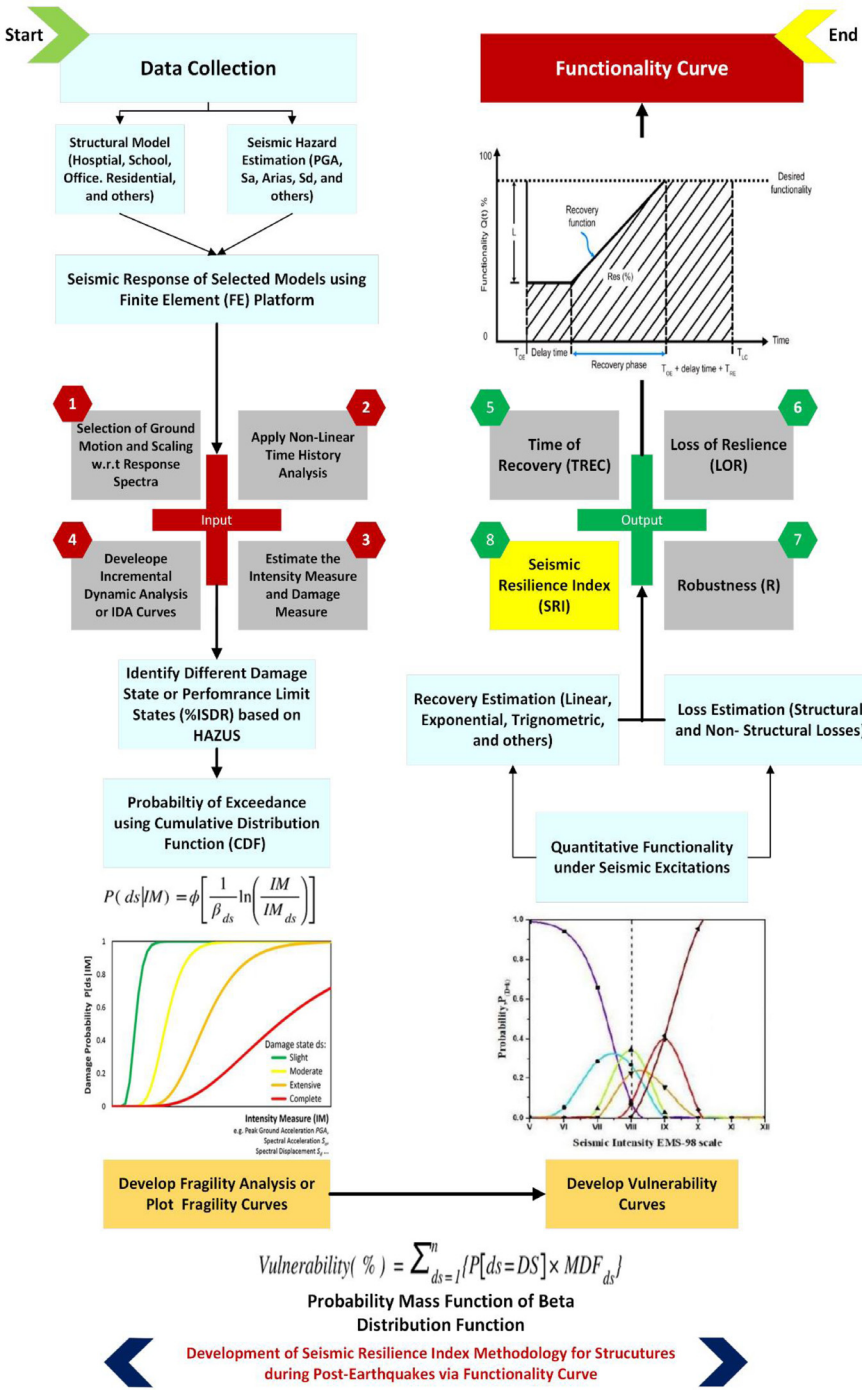
Development of Seismic Resilience Index Methodology Framework.

By reviewing the previous research, most of the researchers adopted a similar methodology for deriving the seismic resilience index. The method and flow for the evaluation of the seismic resilience index of the building are clearly shown in Fig. 2. The following represents the procedures of the proposed research methodology.

Step 1: Collect and compile the relevant information for the selected RC buildings.
Step 2: Modelling the structure of the case study by utilizing the Finite Element (FE) platform (e.g., ETABS)
Step 3: Identifying the criteria of strong ground motion records selection from far-field and near-field seismic scenarios to obtain the non-linear dynamic analysis (NL-DA).
Step 4: Scaling the strong ground motion records with respect to a particular response spectrum according to Malaysia National Annex 2017.
Step 5: Determining the performance limit states (PLS) to be used in the research.
Step 6: Identifying the damage measure (DM) and intensity measure (IM) to be used in the research.
Step 7: Performing the non-linear dynamic approach to generate the Incremental Dynamic Analysis (IDA), for each strong ground motion record.
Step 8: Determining the probability of reaching or exceeding a performance limit state (PLS) by using the fragility function where the mean collapse intensity and the standard deviation can be obtained throughout the IDA.
Step 9: Plotting the fragility curve for the given performance limit states.
Step 10: Assessing the vulnerability curves for each performance limit state by extracting all the discrete probability of performance limit states obtained from the fragility curves.
Step 11: Assessing the direct loss function of the case study under the far-field and near -field seismic scenarios.
Step 12: Estimating the recovery time of the case study under the far-field and near -field seismic scenarios using the Linear Time Recovery Function.
Step 13: Develop the functionality curve for the case study using the parameters (direct loss and recovery time) and indicating the functionality of the case study from 0 to 1.
Step 14: Determining the Seismic Resilience Index (SRI) for the case study under the seismic scenario from the functionality curve.
Step 15: Evaluating the robustness of the case study under the seismic scenario.
Step 16: Schematizing the Seismic Resilience Index (SRI) Map for each building cluster using the Geographical Information System (GIS) platform for far-field and near-field seismic scenario.

### Building application case studies-damaged during Ranau earthquake 2015

The most recent major and strongest earthquake that happened in Malaysia is the 2015 Sabah Earthquake which struck Ranau with a magnitude of 6.0 on 5th June 2015. Intending to protect the structures against the hits of the earthquake, the resistance of structures in Malaysia against the seismic has to be concerned and improved. During the 2015 Sabah Earthquake, the reinforced concrete school and hospital buildings had been reported damaged. Thus, in this study, a 4-storeys reinforced concrete school building and a 5-storeys reinforced concrete hospital building are selected as the target prototype sample for this approach. Furthermore, a total of 30 existing reinforced concrete buildings in Malaysia are chosen as the target of the research for GIS mapping purpose. The architectural drawings of the chosen structural models with different dimensions are acquired in advance for modelling and analysis purposes. The design codes used for the analysis are Eurocodes such as Eurocode 1, Eurocode 2, and Eurocode 8. The input design code follows the codes of practice used in the design of the buildings. Similarly, the input parameters such as loadings and properties of materials are identified based on the design codes.

(a) Reinforced Concrete School Building

**Fig 3:**
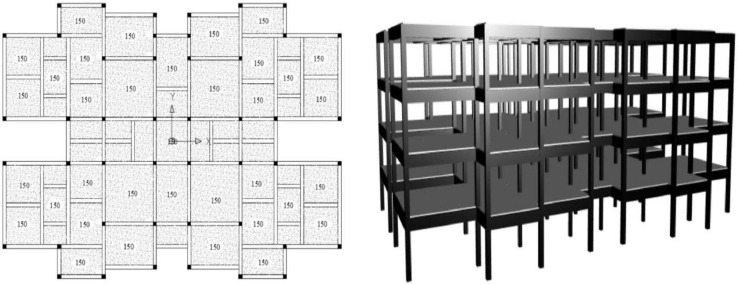
Plan view and 3D view of the 4-storey RC school building [17].

**Fig 4:**
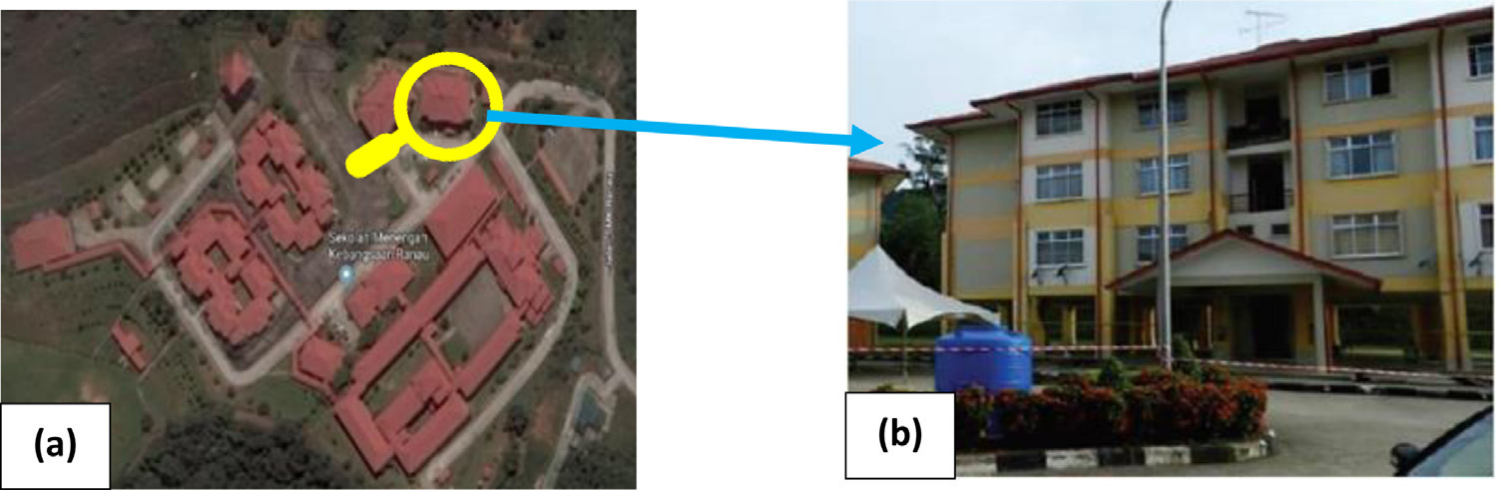
(a) Location of the school building on the map, (b) photography of school building [17].

A 4-storey reinforced concrete school building with a total building floor area of 1899 m^2^ and a slab thickness of 150mm for all stories was selected as the first model in this study to apply the proposed research methodology. The mainframe of the building is formed by reinforced concrete columns, beams, slabs, and infill brick walls. Additionally, the RC school building was built on soft clay soil which is classified as soil type D [17]. The plan view and the 3D view of the building illustrated in Fig. 3 had shown that the building was identically in plan and elevation. The location of the school building on the map and the photography of the school building is shown in Fig. 4. The structure was designed in accordance with Eurocodes with the material properties for all the structural elements as shown in Table 1. Furthermore, the dimensions and the reinforcements of the columns and beams of the RC school building, as well as the detailing of the reinforcement are tabulated in Tables 2 and 3, respectively.

(b) Reinforced Concrete Hospital Building

**Table 1:** Material properties of the RC building.

Material	Unit Weight (kN/m^3^)	Characteristic Strength (MPa)	Young's Modulus (MPa)
Concrete	25	30	31,000
Steel Reinforcement	77	500	200,000

**Table 2:** Dimensions and reinforcement detailing of columns [17].

Column Properties	1^st^ Storey	2^nd^ Storey	3^rd^ Storey	4^th^ Storey	Detailing Section
Dimension (mm)	300 × 300	300 × 300	300 × 300	300 × 300	
Rebars	8H20	8H16	8H14	8H12	
Shear links	H10-300	H10-300	H10-300	H10-300	
Dimension (mm)	250 × 250	250 × 250	250 × 250	250 × 250	
Rebars	4H20	4H16	4H14	4H12	
Shear links	H10-300	H10-300	H10-300	H10-300

**Table 3:** Dimensions and reinforcement detailing of beams [17].

4-storey RC School Building			
Dimension (mm)	300 × 600	250 × 550	250 × 600
Top Rebars	3H12	2H12	3H12
Bottom Rebar	6H12	3H16	3H12
Shear links	H10-200	H10-250	H10-250
Detailing section			

**Fig 5:**
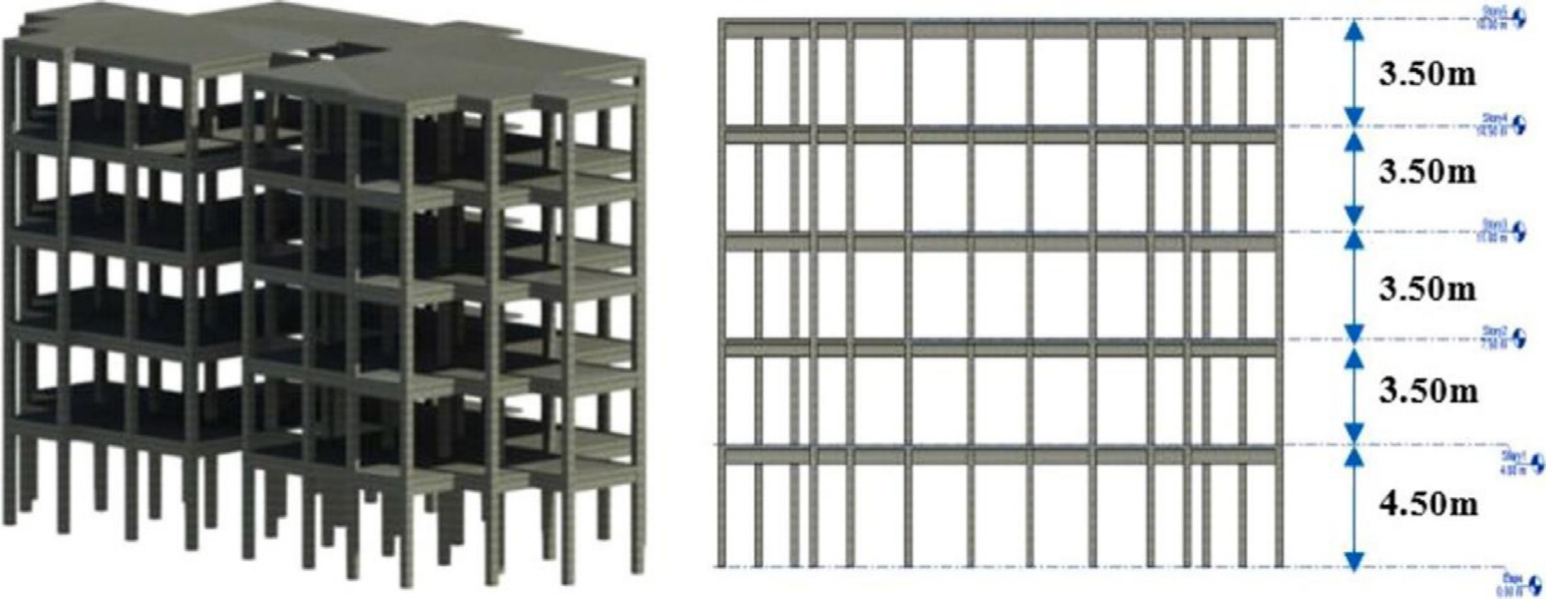
3D view and elevation view of the 5-storey RC hospital building [18].

**Fig 6:**
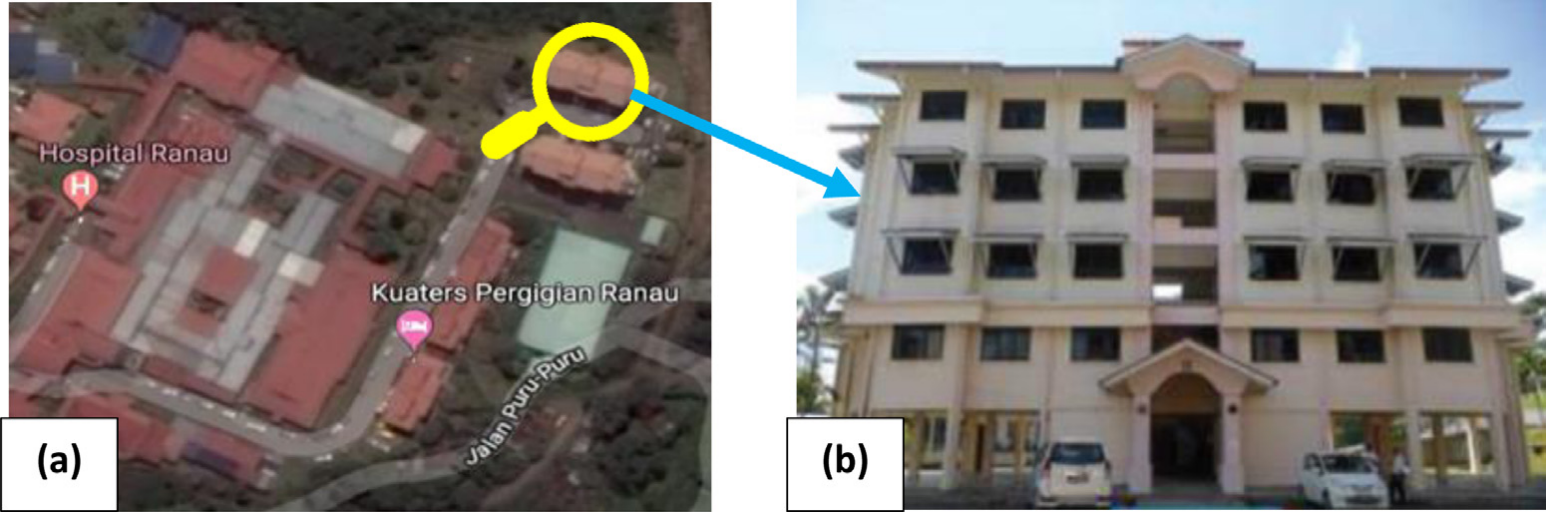
(a) Location of hospital building on the map, (b) photography of hospital building [18].

A 5-storey reinforced concrete hospital building with a 4500mm height for ground level and 3500mm for the following levels was selected as the second model in this study to be applied for this research method. The mainframe of the building is formed by reinforced concrete columns, beams, slabs with a uniform thickness of 150mm for all stories, and infill brick walls. The 3D view and the elevation view of the RC hospital building are illustrated in Fig. 5. The location and the photography of the RC hospital building are shown in Fig. 6. Similarly, the structure was designed in accordance with Eurocodes. Furthermore, the dimensions and the reinforcements with detailing sections of the columns and beams of the RC hospital building are tabulated in Table 4.

**Table 4:** Dimensions and reinforcement detailing of columns and beams [18].

Frame Sections	Beam		Column	Beams Detailing	Columns Detailing
Size (mm)	250 × 600		350 × 350		
Reinforcement	Top	Bottom	12H12		
Number of bars	3H12	3H12			
Shear Links	H8-300		H8-300		

### Building data collection

In this study, in order to assess the seismic resilience index of existing buildings in Malaysia and present the results visually using the Geographical Information System (GIS) platform, the data and information of a total of 25 existing reinforced concrete (RC) buildings located in Penang are collected. The buildings are then categorized into three different clusters which are low-rise buildings (1 to 3-storeys), mid-rise buildings (4 to 7-storeys), and high-rise buildings (8-storeys and above). The selected buildings comprised four major occupancy classes, namely residential, commercial, hospitals, and schools.

**Fig 7:**
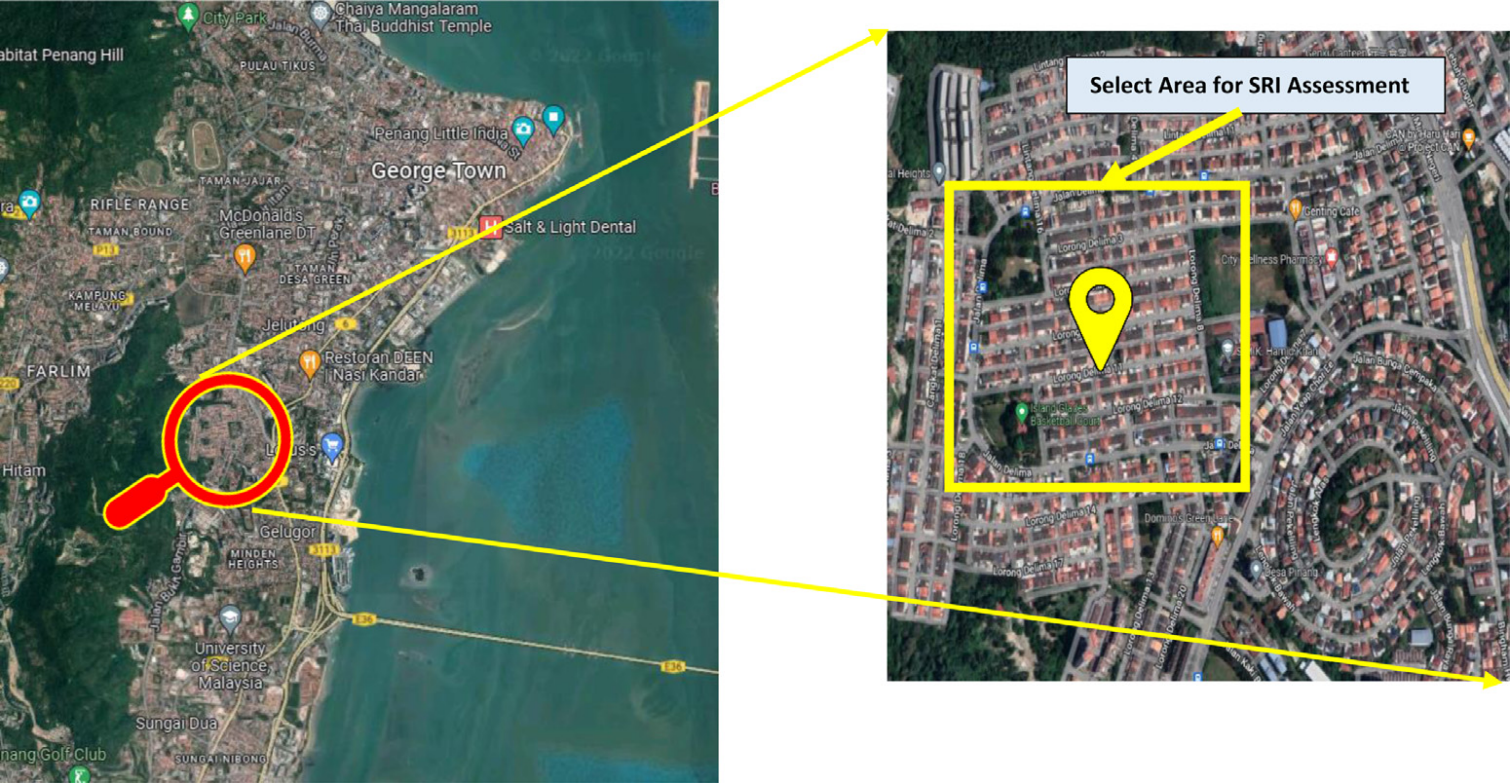
Location of selected buildings on the map.

The geometric layouts of the targeted buildings are compiled in CAD files for modelling and analysis purposes to employ the methodology of the seismic resilience index. The buildings have a similar mainframe which is configured by RC columns, RC beams, RC slabs, and infill brick walls. All the selected RC buildings are presumed to have an identical height for each storey with variable width bay and slab thickness in the range of 150mm to 200mm. The location of the chosen buildings is illustrated in Fig. 7. Furthermore, the structural properties and the 3D views of the low-rise, mid-rise, and high-rise buildings are summarized in Tables 5, 6, and 7, respectively.

**Table 5:** Structural Properties of the chosen low-rise buildings.

Building ID	No. of Storey	Building Height (m)	Column Size (mm)	Longitudinal Reinforcement	Beam Size (mm)	3D view
BLR-1	3	10.00	400 × 400	8H16	250 × 600	
BLR-2	2	6.00	250 × 250	4H16	200 × 500	
BLR-3	2	6.00	200 × 150	4H10	200 × 500	
			300 × 300	8H12		
BLR-4	2	7.00	400 × 400	8H16	300 × 500	
BLR-5	2	7.20	300 × 300	8H12	200 × 450	
BLR-6	2	7.00	300 × 300	8H12	150 × 450	
BLR-7	2	6.50	200 × 200	4H10	200 × 500	
			150 × 150	4H10		
BLR-8	2	6.50	350 × 450	8H16	250 × 350	
BLR-9	2	7.40	450 × 450	8H20	200 × 450	
BLR-10	2	6.50	200 × 200	4H10	200 × 400	
BLR-11	2	4.00	250 × 250	6H12	250 × 500	
BLR-12	2	9.50	200 × 450	8H12	250 × 500

**Table 6:** Structural Properties of the chosen mid-rise buildings.

Building ID	No. of Storey	Building Height (m)	Column Size (mm)	Longitudinal Reinforcement	Beam Size (mm)	3D view
BMR-1	4	12.00	350 × 200	6H12	200 × 400	
			200 × 400	4H12		
			400 × 250	5H12		
BMR-2	4	12.00	400 × 300	6H16	300 × 500	
BMR-3	4	12.00	400 × 600	8H20	400 × 600	
BMR-4	5	16.00	150 × 300	4H12	150 × 300	
BMR-5	4	14.40	450 × 250	6H16	250 × 500	
			250 × 250	4H16		
BMR-6	4	12.50	400 × 400	8H16	300 × 600	
			400 × 300	6H16		
BMR-7	5	18.00	200 × 200	4H12	200 × 600	
			200 × 450	5H16		
			450 × 150	6H12

**Table 7:** Structural Properties of the chosen high-rise buildings.

Building ID	No. of Storey	Building Height (m)	Column Size (mm)	Longitudinal Reinforcement	Beam Size (mm)	3D view
BHR-1	9	27.00	500 × 500	8H20	250 × 500	
BHR-2	10	34.00	300 × 500	5H20	250 × 450	
			250 × 450	6H16		
BHR-3	9	28.80	400 × 650	6H25	200 × 600	
BHR-4	9	27.00	300 × 300	8H12	250 × 600	
BHR-5	11	33.00	500 × 250	7H12	300 × 500	
			700 × 250	8H20		
BHR-6	8	20.60	250 × 450	6H16	150 × 450	
			250 × 400	6H16		
			250 × 600	5H16		

### Damage observations

**Fig 8:**
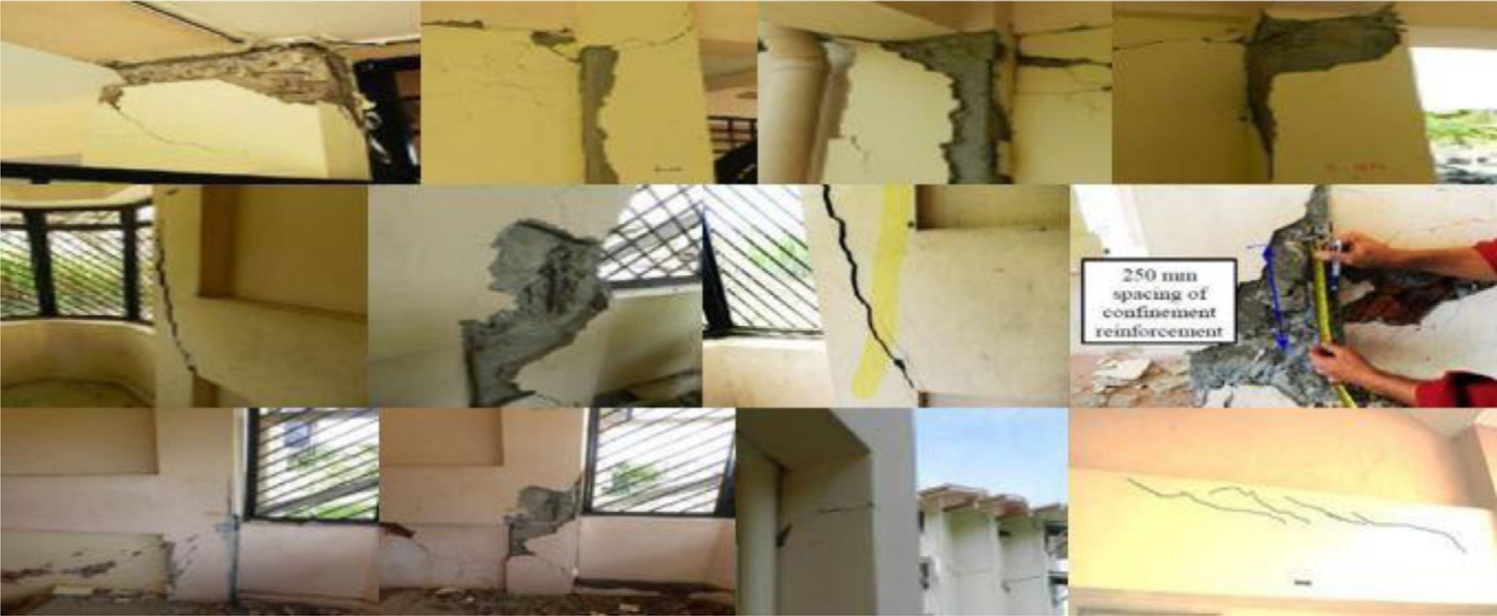
RC beam-column joints and columns damages due to 2015 Ranau earthquake in Hospital Building [19].

As can be seen in Fig. 8, Dora et al. [19] conducted visual inspections of the Ranau Hospital building after the earthquake that occurred in 2015. This demonstrates that the majority of the damage took place at the joints between the beams and the columns, whereas the majority of the damage found at the ends of the columns was concentrated at the ground level of the structure.

**Fig 9:**
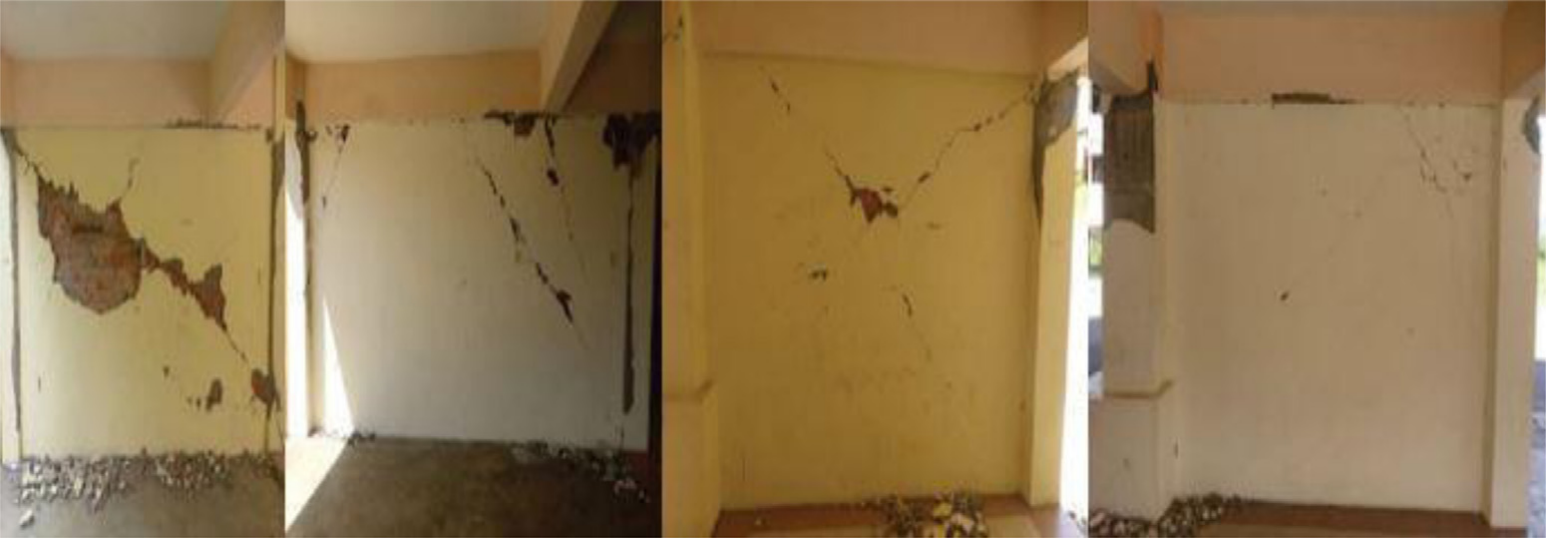
In-plane wall failure due to the 2015 Ranau earthquake in Hospital Building [19].

The consequences of rapid evaluations show that the distribution of confinement or transverse reinforcements is insufficient for withstanding the loads that are caused by earthquakes. In addition, non-structural materials like masonry walls have a propensity to have an in-plane failure mechanism that is reflected in sheared-off x-diagonal cracks due to the brittle failure of the material. This is illustrated in Fig. 9. As a result, it was discovered that soft-storey buildings are unable to withstand earthquake loads, which can result in major damage as well as the collapse of the structure. Even if the damage reaches the zone where the column becomes plastic, the beams may continue to exist in the elastic zone.

**Fig 10:**
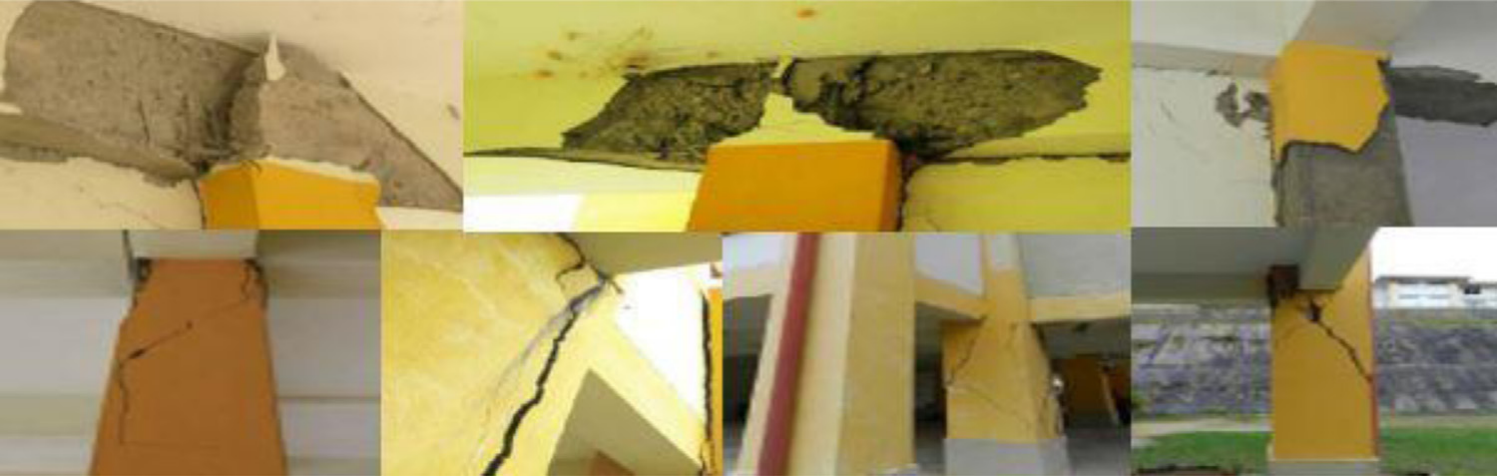
Significant damages on RC beam-column joints and on RC-columns due to 2015 Ranau earthquake for School Building [20].

Moreover, and according to Fig. 10, Dora et al. [20] conducted visual observations of the School building after the earthquake. According to the findings of the investigation, the most of the damage happened on the ground floor, and more specifically in the crucial zone, which consists of the beam-column joins and the column itself. According to the preliminary analysis of the structural damage, it is fairly evident that the vertical elements are unable to withstand the lateral loads that are being applied. As a consequence of this, the columns sustained significantly more damage than the RC beams. In contrast to the beams, which are still in the elastic zone, the columns play an essential part in carrying all of the shear forces that are created by the lateral load.

### Selection of strong ground motion and performance limit states

The next essential and critical step in defining the seismic load input for the structural analysis is the selection of the strong ground motion records. The parameters that influence the selection of strong ground motions include the magnitude range of the anticipated significant events, the distance range of the site from the causative faults, the peak ground acceleration, and the soil types. Thus, these parameters must be considered in the selection of the strong ground motion records.

**Fig 11:**
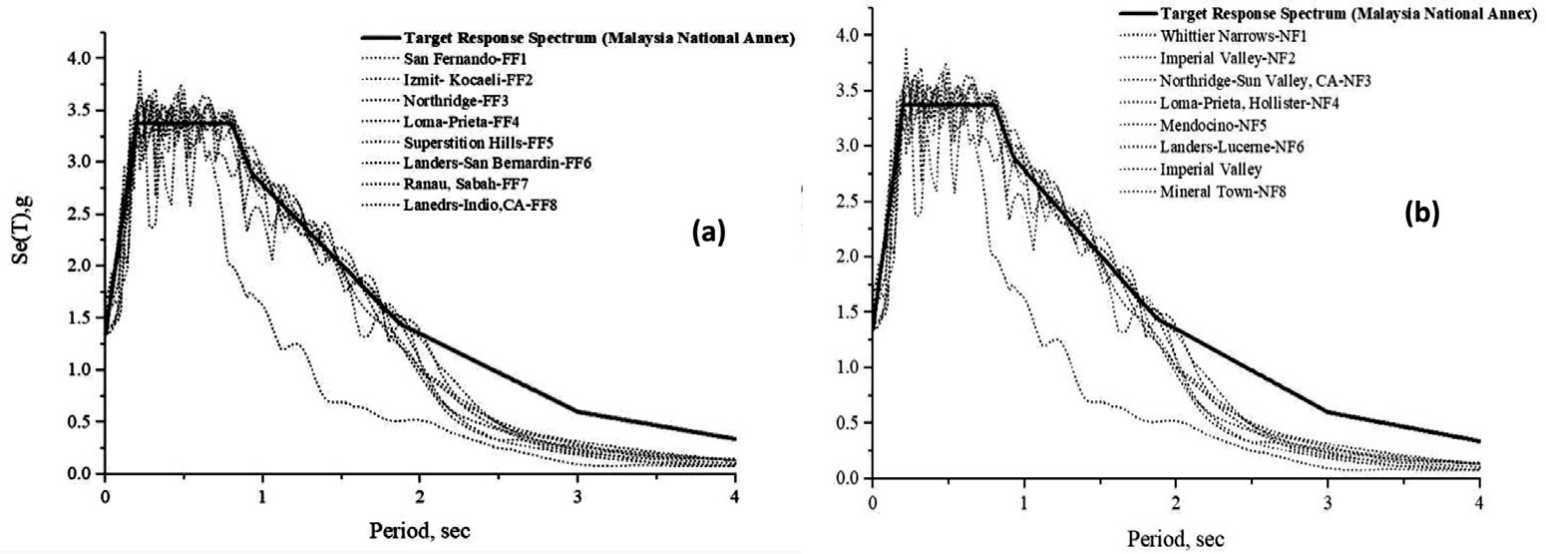
Scaling ground motion with respect to the target response spectrum (a) Far-Field, and (b) Near-Field [18].

In this research, the methodology of code-based record selection is adopted. The strong ground motion records are selected based on the regulations and the recommendations of Eurocode 8 (EC8). By taking into consideration the regulations and the recommendations of the design code, as well as the recommendation made by the majority of the codes to have a minimum of three to seven sets of input seismic records, whereas a set of 15 strong ground motion records that are clustered between far-field and near-field are selected and scaled in order to obtain the mean response spectrum to be used in the simulation analysis, the regulations and the recommendations of the design code are taken into consideration. The characteristics that have an influence on the selection of strong ground motions are taken into consideration, and the strong ground motions are chosen according to the criteria that are outlined in Table 8. Nevertheless, the strong ground motions are scaled with regard to the target design spectrum, which comprises the code spectrum, the uniform hazard spectrum, and the condition mean spectrum. This spectrum is utilized to reflect the demand of the structure design [21]. In which the design response spectra for Sabah-Ranau were implemented for the selected Ranau structures (the School and the Hospital), and ground type B was taken into consideration by referring to Takano and Saito [22]. The parameters recommended for the Sabah elastic response spectrum are as follow (S=1.40, T_B_=0.15, T_C_=0.4, and T_D_=2.0). In accordance with the Malaysia National Annex 2017, the selected ground motion records for near-fault and far-fault are scaled with respect to the target response spectrum as shown in Fig. 11.

**Table 8:** Parameters of strong ground motion for the selection criteria.

Fault Type	All Type
Magnitude	5.0 to 8.0
Distance To Surface Projection of The Rupture (RJB)	≤ 20km (Near-Fault) > 20km (Far-Fault)
Soil Profile Type	D (clay soils)
Shear Wave Velocity	180 m/s to 360 m/s
Pulse Type	All Type

Evidently, a variety of Peak Ground Acceleration (PGA) intensities must be considered. All the selected records are rather broad since their PGA ranges among 0.125g and 0.684g and their magnitude (Mw) varies from 5 to 8. Most tremors felt in Malaysia have these intensities.

With the targeted criteria, every 15 sets of ground motion data for near-fault and far-fault are downloaded from the Consortium of Organizations for Strong-Motion Observation System Database (COSMOS) as shown in Tables 9 and 10, respectively.

**Table 9:** Selected near-fault ground motion records from the COSMOS database [18].

No.	Event	Station	Year	PGA (g)	Distance (km)	Magnitude (Mw)
1	Whittier Narrows	Los Angeles, CA	1987	0.182	19.50	6.10
2	Imperial Valley	EL Centro, CA, Irrigation District	1940	0.341	12.20	6.90
3	Northridge	Sun Valley, CA	1994	0.457	10.50	6.70
4	Loma-Prieta	Hollister, CA	1999	0.252	16.80	6.94
5	Mendocino	Cape Mendocino, CA	1992	1.400	0.00	7.01
6	Landers	Lucerne	1992	0.650	2.19	7.28
7	Imperial Valley	EL Centro Array #7	1940	0.340	0.56	6.53
8	Mineral Town	Central Virginia	2011	0.094	18.53	5.74

**Table 10:** Selected far-fault ground motion records from the COSMOS database [18].

No.	Event	Station	Year	PGA (g)	Distance (km)	Magnitude (Mw)
1	Izmit-Kocaeli	Nuclear Research Centre	1999	0.181	101.10	7.40
2	Landers	San Bernardino, CA	1992	0.332	79.60	7.28
3	Superstition Hills	Calipatria, CA	1987	0.252	27.00	6.54
4	Loma-Prieta	Emeryville, CA	1989	0.490	67.70	7.00
5	Northridge	Santa Monica, CA	1994	0.684	28.20	6.69
6	Ranau, Sabah	KKM_HNE	2015	0.125	60 to 70	6.10
7	Landers	Indio, CA	1992	0.302	49.10	7.30

Furthermore, the performance limit states adopted in this research are according to the most common methodology of HAZUS which defines the instance probability damage of a structure into four damage states: Slight, Moderate, Extensive, and Complete. The damage measure (DM) used in this work is drift whereas, drift ratios of 0.05, 0.10, 0.15, and 0.20 are adopted to represent the aforementioned damage states respectively. For reinforced concrete moment-resisting frames, the HAZUS-MH 2.1 [23] appendix provides explanations for each of the four limit states are listed in Table 11, and the definition of the performance limit states are as defined in the technical manual.

**Table 11:** Definition of the performance limit states [23].

Performance Limit State (PLS)	Inter-Storey Drift Ration (ISDR)	Description
Slight	0.5%	Some beams and columns have flexural or shear type microcracks around connections
Moderate	1.0%	Most beams and columns are cracked. Larger flexural cracks and some concrete spalling can be seen in ductile frames that have reached their yield capability. Larger shear splits and spalling can be seen in nonductile frames.
Extensive	1.50%	Large flexural fractures, spalled concrete, and buckled primary reinforcement indicate some frame parts have reached their full capacity in ductile frames; Shear or bond failures at reinforcement splices, broken links, or buckled primary reinforcement in columns might cause sudden collapse in non-ductile frames.
Complete	2.0%	The structure has collapsed or is about to collapse owing to brittle frame failure or frame instability. Approximately, Low-rise buildings are predicted to lose around 13% of their reinforced concrete moment resistant frames (RC-MRF), while mid-rise buildings will lose 10%, and high-rise structures will lose 5% of the RC-MRF

### Incremental dynamic analysis

As described by Plumbridge et al. [24], a non-linear analysis is an extension of linear analysis which is an incremental-iterative process that is based on the techniques of matrix solution and adopts elements, material models, loadings, and boundary conditions. In finite element analysis (FEA), the non-linear analysis plays a critical role in simulation to understand the non-linearity of the model such as material non-linearity (elasticity, plasticity, elasto-plasticity, and creep deformation), geometric non-linearity (large deflection and large strains), and constraint and contact non-linearity (stiffness changes during load events) (Grasp Engineering, 2020). There are two major types of non-linear analysis, namely non-linear static pushover analysis (NL-SA) and non-linear time history dynamic analysis (NL-DA).

Incremental Dynamic Analysis (IDA) uses a sequence of non-linear dynamic analyses under a suite of strong ground motion records to anticipate seismic demand and capacity [25]. IDA is applied widespread due to some of its objectives: full understanding of the responses or earthquake demand of the structure in a wide range of different levels of seismic records; better understanding of the structural effects at different seismic levels with less or more power; better understanding of changes of the nature of structure responses with the increase of the seismic intensity; and evaluation of the dynamic capacity of the entire structural system.

According to Arshadi [26], the results of IDA in comparison to the other types of analyses are closer to the reality of the structural behavior due to the dynamic and non-linear nature of the earthquake. However, the drawback of this method is time-consuming and is highly dependent on the number of records and data input. As defined by Vamvatsikos and Cornell [25], plotting a damage measure (DM) recorded in an IDA study against one or more intensity measures (IMs) that represent the applied scaled accelerogram produces an IDA curve. Depending on the amount of intensity measures used, an IDA curve can have two or more dimensions.

Intensity measures (IMs) are used to connect engineering demand parameters (EDPs) to seismic hazard analysis results. Moreover, an optimum intensity measure has four desired features: efficiency, sufficiency, scaling robustness, and predictability [27]. According to Shome et al. [28], spectral acceleration (Sa) served as a more consistent intensity measure and can be quantified with Ranau seismic intensity measure of (VIII) in the evaluation of incremental dynamic analysis, especially for a structure that can be represented by a single-degree-of-freedom system. Moreover, the Damage measure (DM), also known as structural state variable, is an observable quantity that can be derived from the output of the corresponding incremental dynamic analysis. As explained by Vamvatsikos and Cornell [25], the maximum joint or node rotation will be the most effective damage measure for the evaluation of structural damage of a frame building compared to other parameters. Thus, in this research, the parameter that is used as a damage measure is the node rotation which can be defined by the maximum inter-storey drift ratio (ISDR).

Incremental dynamic analysis (IDA) also known as the non-linear dynamic (NL-DA) approach depends on the parametric analysis method used in performance-based earthquake engineering (PBEE) to evaluate the structural performance under seismic events [13,29]. It is mandatory to perform incremental dynamic analysis for the structures to extract the seismic resilience index (SRI). The incremental dynamic analysis is carried out under each strong ground motion record and the accelerations with different scale factors and time steps are defined. In addition, a Rayleigh damping of 5% is considered in the analysis as per described in Eurocode 8. Thus, the elastic response spectra are incrementally developed from 0.1g a until the structural model reaches the collapse state. The IDA curves can be developed by plotting the variation of the node rotation as a function of the spectral acceleration (Sa) where the spectral acceleration (Sa) is the intensity measure (IM), and the maximum inter-storey drift ratio (ISDR) is the damage measure (DM).

### Fragility and vulnerability assessment using probabilistic approach

**Fig 12:**
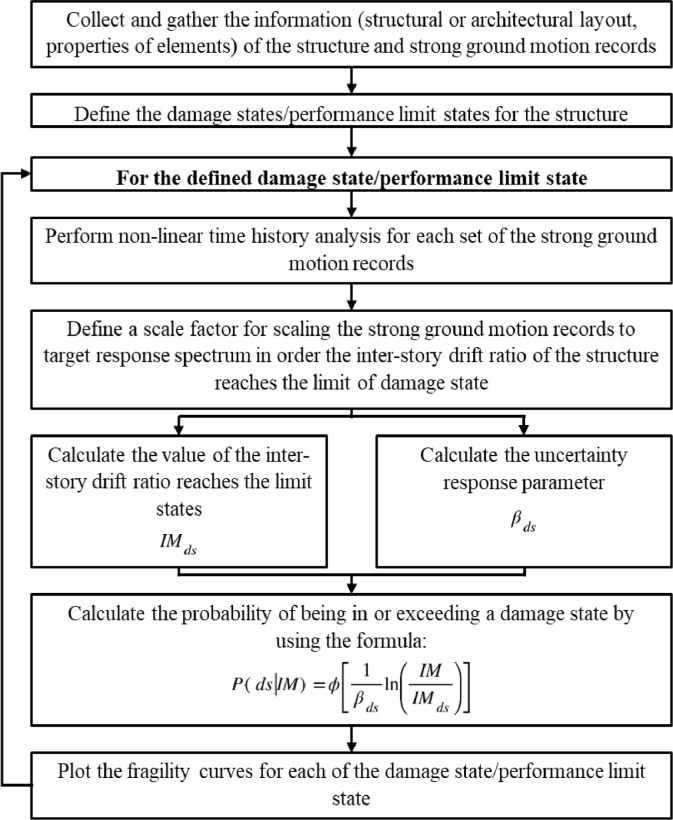
Fragility curve development flowchart using cumulative distribution function.

In the perspective of earthquake engineering, the fragility curve is usually acted as a tool for the quantification function of some measure of the environmental excitation. Several parameters can be used for the assessment of fragility curves such as peak ground acceleration (PGA), spectral acceleration (Sa), spectral displacement (Sd), and peak ground velocity (PGV). Fig. 12 provides an illustration of the processes that have been taken in general to evaluate the fragility curve.

The fragility curves are defined in terms of two basic parameters which are the mean collapse intensity and the standard deviation. Hence, as represented by Shinozuka et al. [30] and Hosseinpour et al. [31], the function used is expressed as Eq. (1), which is found to be suitable for use in most structural types.

(1) $$P\left(ds|IM\right)=\phi \left[\frac{1}{\beta_{ds}}\ln\left(\frac{IM}{IM_{ds}}\right)\right]$$

Where:

IM is the intensity measure such as spectral acceleration (Sa),

$IM_{ds}$ is the value of intensity measure at which the structures reach the limit state threshold value,

$\beta_{ds}$ is the uncertainty response parameter,

ϕ is the standard normal cumulative distribution function,

To develop a “better estimate” and “higher accuracy” fragility curve, a combined random variable term should be used as the uncertainty of each damage state is not practically considered to be separated from the randomness [32]. The uncertainty response parameter can be modelled by the combination of three major contributors to damage variability: the model, the capacity, and the demand uncertainty, which is obtained in Eq. (2).

(2) $$\beta_{ds}=\sqrt{{\left[\left(CONV\left[\beta_{c},\beta_{d}\right]\right)\right]}^{2}+{\left[\beta_{M(ds)}\right]}^{2}}$$

Where:

$\beta_{M(ds)}$ is the uncertainty associated with the limit state threshold values and commonly taken to be 0.4 in HAZUS,

$\beta_{M(ds)}$ is the uncertainty associated with the building capacity,

$\beta_{d}$ is the uncertainty associated with imposed earthquake demand which is taken to be 0.45 at short periods and 0.5 at long periods in HAZUS,

Seismic vulnerability curves can be used to obtain the loss function which is an important parameter to evaluate the seismic resilience index (SRI). The vulnerability curve specified the probability of damage of given damage state under certain seismic intensity. The most common and popular method to develop vulnerability curves is transforming directly from the appropriate fragility curves of the structures, in another way, in another word, it is a direct use of probability mass function (PMF) rather than cumulative distribution function (CDF). In this approach, the vulnerability curves are developed from fragility curves by combining all the discrete probability of performance limit state. The vulnerability curves can be determined using Eq. (3).

(3) $$\text{Vulnerability}\left(\%\right)=\sum_{ds=1}^{n}\{P\left[ds=DS\right]\times MDF_{ds}\}$$

Where *MDF_ds_* is the mean damage factor which is the central value of the damage factor range. The damage factor range can be adapted from the respective methodology of limit states. Table 12 shows the damage factor range adapted from the methodology of HAZUS and the mean damage factors applied in this research.

**Table 12:** Damage states with the corresponding damage factor ranges adapted from HAZUS and the mean damage factors applied [33].

Damage State	Damage Factor Range (%)	Mean Damage Factor (%)
None	0	0
Slight	0 – 4	2
Moderate	4 – 16	10
Extensive	16 – 84	50
Complete	100	100

### Assessment of seismic resilience index

Seismic resilience is a concept to evaluate the post-earthquake functionality of structures that significantly play a critical role in post-earthquake rescue and recovery. Generally, the seismic resiliency is evaluated in percentage and varies from 0% to 100% where 0% represents spectacular failure and 100% represents no structural failure or damage. In order to develop the seismic resilience index, the resiliency of the structures is normalized from 0 to 1.

**Fig 13:**
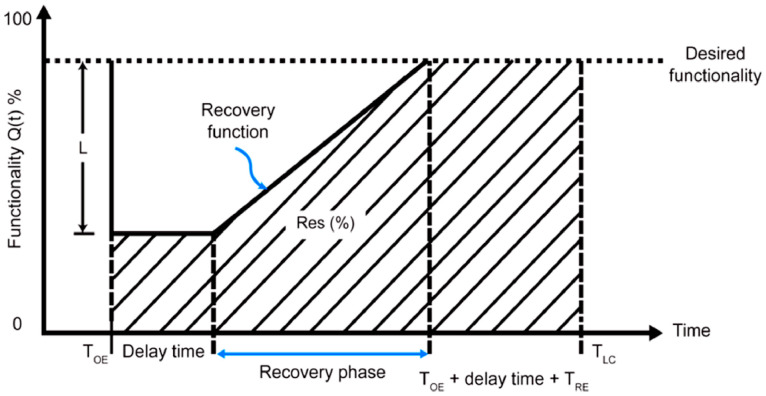
Example of functionality curve [12].

Seismic resilience is defined as the functionality of a structure after the recovery process or the ability of a structure to withstand earthquakes without any reduction in its functionality [2]. Therefore, by referring to the functionality curve as shown in Fig. 13, the seismic resilience index can be determined.

The seismic resiliency of the structures is demonstrated by the area under the functionality curve which can be determined by the equation expressed as Eq. (4) [5]:

(4) $$R=\frac{1}{T_{LC}}\int_{t_{OE}}^{t_{OE}+T_{LC}}Q(t)dt$$

Where;

$T_{LC}$ is the control time interested which is the investigated time interval after an earthquake (usually considered to be 50 years for residential buildings) or the longest recovery time under the considered seismic intensities.

$t_{OE}$ the time when the event happens, or the time earthquake shock happens which is usually taken as 0.

Q(t) the dimensionless percentile which is defined as the functionality function.

### Development of functionality curves

The percentage of a structure's functionality across the control period is defined by the functionality curve. The functionality function, which is a non-dimensional quality system function, is used in the development of the functionality curve. Several factors, such as the amount of direct loss, the amount of indirect loss, and the recovery time, can be used to evaluate the functionality of a building. The functionality function is expressed as Eq. (5). Due to the function's stated goal of 100% functioning, the function's output values are less than 1 for all quantities.

(5) $$Q\left(t\right)=\left[1-L(1,T_{RE})\right]\cdot \left\{H\left(t-T_{OE}\right)-H\left(t-\left[T_{OE}+T_{RE}\right]\right)\right\}\cdot f_{REC}\left(t,T_{OE},T_{RE}\right)$$

Where;

$L(I,T_{RE})$ is the loss function, $f_{REC}\left(t,T_{OE},T_{RE}\right)$ is the recovery function, H(t)is the Heaviside step function, $T_{RE}$ is the recovery time after an event; and, $T_{OE}$ is the time of the occurred event with intensity I.

### Direct loss function

For most cases, the loss function is a composite of direct and indirect losses. Whereas structural and non-structural losses are classified as "direct losses," temporal dependencies are classified as "indirect losses.". The loss function is expressed as Eq. (6).

(6) $$L\left(1,T_{RE}\right)=L_{D}+\left(\alpha_{i}\cdot L_{i}\right)$$

Where $L_{D}$ and $L_{I}$ represent the direct losses and indirect losses, respectively, whereas $\alpha_{I}$ is the weight factor that depends on the significance of the structures for the society and the influence of the structure on the other system. However, the indirect losses are neglected in this research.

The direct loss function can be efficiently determined by making the use of vulnerability curves, which are adequate for calculating the percentage of estimated degree of damage to a structure. In addition, Samadian et al. [13] led to the realization that the results of resiliency derived from the vulnerability curves are superior to those retrieved from the fragility curves in terms of their ideal quality. Consequently, the vulnerability curves can be utilized in this research methodology to perform an evaluation of the loss function by employing the modified formula that is presented in Eq. (7) [6].

(7) $$L_{DE,K}\left(I\right)=\left[\frac{C_{s}}{I_{s}}\cdot \prod_{i=1}^{t_{i}}\frac{1+\delta_{i}}{1+r_{i}}\right]\cdot Damage\left(\%\right)$$

Where, $C_{s}$ is the building repair cost, $I_{s}$ is the replacement building costs, $\delta_{i}$ is the annual depreciation rate, $r_{i}$ is the annual discount rate applied for the range in years Damage(%) is the percentage of damage obtained from the vulnerability curve.

### Time recovery function

The recovery process is influenced by a variety of factors, including time and location. The recovery time is the amount of time required to bring back a structure's and infrastructure system's functionality to the point where it can function or provide a similar or higher service than it offered before [34]. According to the explanation given by Cimellaro et al. [35], the linear recovery function is typically utilized in situations in which there is no knowledge regarding the social response. As a result, the linear recovery function will be utilized in this study, and its mathematical representation may be found in Eq. (8).

(8) $$f_{REC}\left(t,T_{OE},T_{RE}\right)=1-\left(\frac{1-T_{OE}}{T_{RE}}\right)$$

Where, $T_{RE}$ is the recovery time after an event, $T_{OE}$ is the time of the occurred event with intensity I, and $t_{OE}$ is the time of earthquake occurred.

### Evaluation of robustness

One of the major concepts that play a critical role in improving seismic resilience is robustness. In earthquake engineering, seismic robustness is defined as the prevention of collapse against strong earthquakes [36]. The robustness index can be expressed in two different ways as Eqs. (9) and (10). Eq. (9) expressed a system that is able to limit the direct consequences to the direct consequences occurring in the initiation phase whereas Eq. (10) expressed the ability of a system to limit the number of failed components to those components which occurred in the initiation phase [37].

(9) $$I_{R_{1}}\left(i\right)=\frac{C_{DI}\left(i\right)}{C_{DI}\left(i\right)+C_{DP}\left(i\right)}$$

(10) $$I_{R_{2}}\left(i\right)=\frac{n_{f,i}\left(i\right)}{n_{f,i}\left(i\right)+n_{f,p}\left(i\right)}$$

Where, $I_{R_{1}}$ is the robustness index, $C_{DI}\left(i\right)$ and $C_{DP}\left(i\right)$is the direct consequences, $I_{R_{2}}$ is the alternative robustness index, and $n_{f,i}\left(i\right)$and $n_{f,p}\left(i\right)$is the number of failed constituents.

### Seismic resilience findings for the target buildings

After performing the nonlinear time history analysis (NL-THA) for far-field and near- field seismic scenario, and the discrete damage probability computed using Eq. (3), subsequently, the seismic resilience index (SRI) and its functionality is developed followed by the direct damage losses (L_D_) and Robustness (R).

In this study, for instance, the amount of time necessary for the recovery process is dependent on the degree of damage sustained by the structure. According to the findings of this research, the amount of time needed for complete recovery should be between the time of the seismic event, which was considered to be the minimum period, and the control time, which was considered to be the maximum period. For the purposes of this investigation, the time of occurrence was treated as though it had occurred on the 30th day of the overall control period of 120 days. Noting that, this work focused on the collapse performance limit state where the damage measure denoted by 2.0% inter-storey drift ratio (ISDR) refer to Table 11.

The two selected damaged building during Ranau are investigated based on the intensity measure of Ranau ground motion of intensity equals to (VIII) equals to 0,13g, peak ground acceleration (PGA). For far-field seismic scenario at intensity measure equals to (VIII) for the school building, the structure losses exceed the 50% by losing a 67% from its robustness, where it robustness equals to 33%, with SRI value equals to 0.67. Besides, the recovery time needed under far-field impact is 46 days under certain retrofitting application to function as before. Similarly for the hospital building, the structure reliance equals to 0.72, with direct physical losses equals to 57%, and robustness 43%. Nevertheless, it needs 28 days to get recovered which is earlier compared to school building at the same seismic intensity measure (VIII).

**Fig 14:**
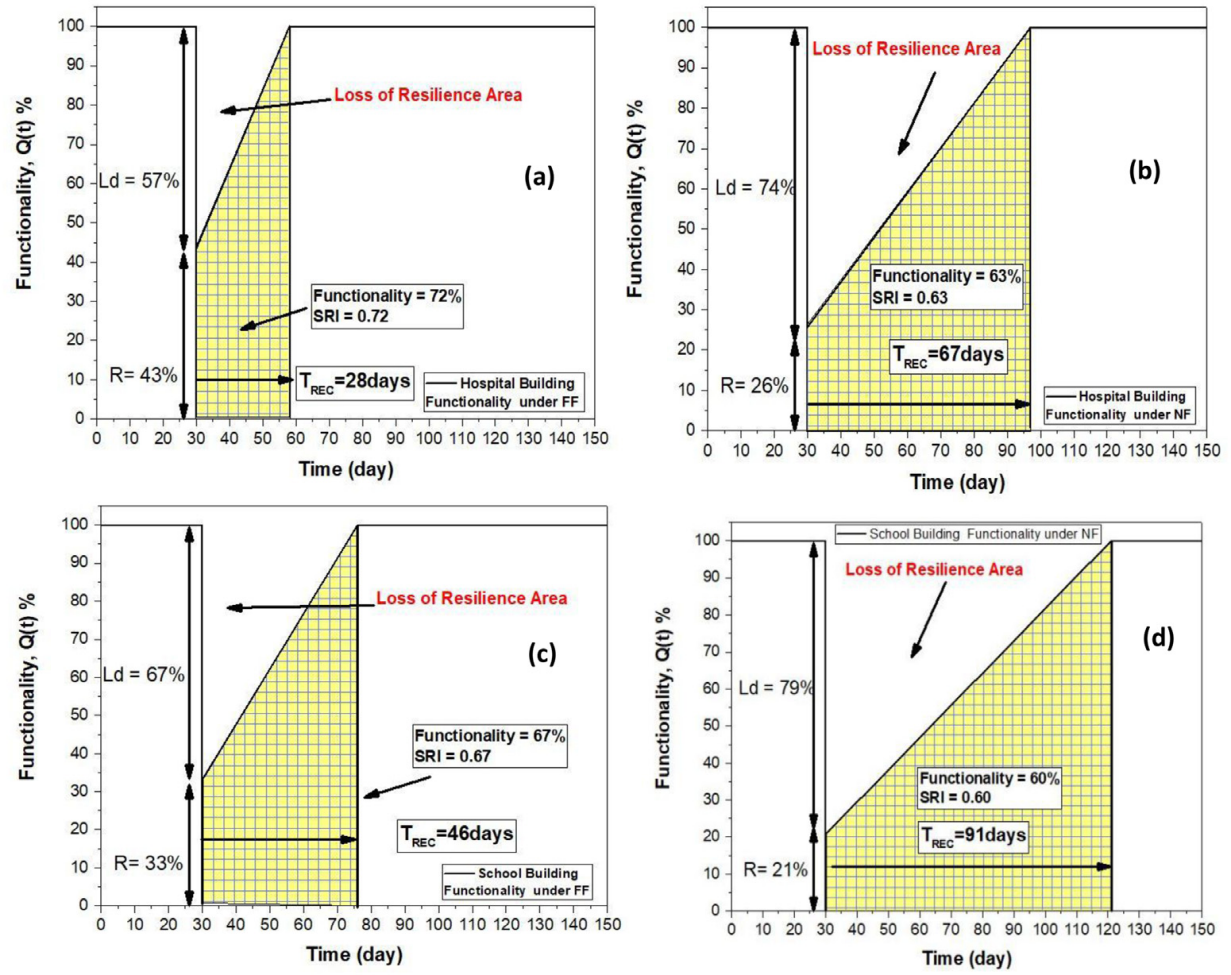
Functionality curves for the selected damaged buildings in case of far-field and near-field seismic scenarios.

On the other hand, in case of near-field seismic scenario, the school building sustains more significant damages compared to far-field scenario. This is demonstrated by the direct losses obtained during near-field impact which reaches 79%, and the amount of time required to recover is 91 days. Similar to the situation with the other targeted hospital building, where the direct losses are also higher in comparison to the far-field influence, which reaches 74%, and where the amount of time required to recover and compensate for the losses equals 67 days. Hence, the % difference between far-field and near field scenarios in terms of direct losses is 50% for school building, and 58% for hospital building, as well the % difference in terms of robustness is 36% for school building, and 40% for hospital building for far-field and near field seismic scenarios, respectively. Moreover, the SRI values a resilience indicator for each target building is reduced within an average of 15% by reaching 0.60, and 0.63 values for school and hospital building, respectively. Hence, both buildings seismic performance behaves similarly in terms of resilience due to the same structural system governed by gravitational loading resistance, and due to the absence of seismic design regulations, this also demonstrated and proved once the seismic vulnerability index (SVI) for each building is developed by Kassem et al. [17,18]. Fig. 14 displays the fluctuation of functionality curve with respect to time by utilizing the linear function. In addition to this, the area that is shaded below the functioning curve reflected the structural system's resistance to seismic activity. Table 13 and 14 summarizes the resilience index, robustness, and losses for each target buildings under far-field and near-field seismic scenarios, respectively.

**Table 13:** Seimsmic resilience assessment of the selected targeted buildings under far-field seismic scenarios.

Seismic Resilience Assessment	School Building	Hospital Building
Seismic Resilience Index (SRI)	0.67	0.72
Time of Recovery (T_REC_)	46	28
Robustness (R)	33%	43 %
Direct Losses (Ld)	67%	57%

**Table 14:** Seimsmic resilience assessment of the selected targeted buildings under Near-field seismic scenarios.

Seismic Resilience Assessment	School Buildings	Hospital Building
Seismic Resilience Index (SRI)	0.60	0.63
Time of Recovery (T_REC_)	91	67
Robustness (R)	21%	26%
Direct Losses (Ld)	79%	74%

**Fig 15:**
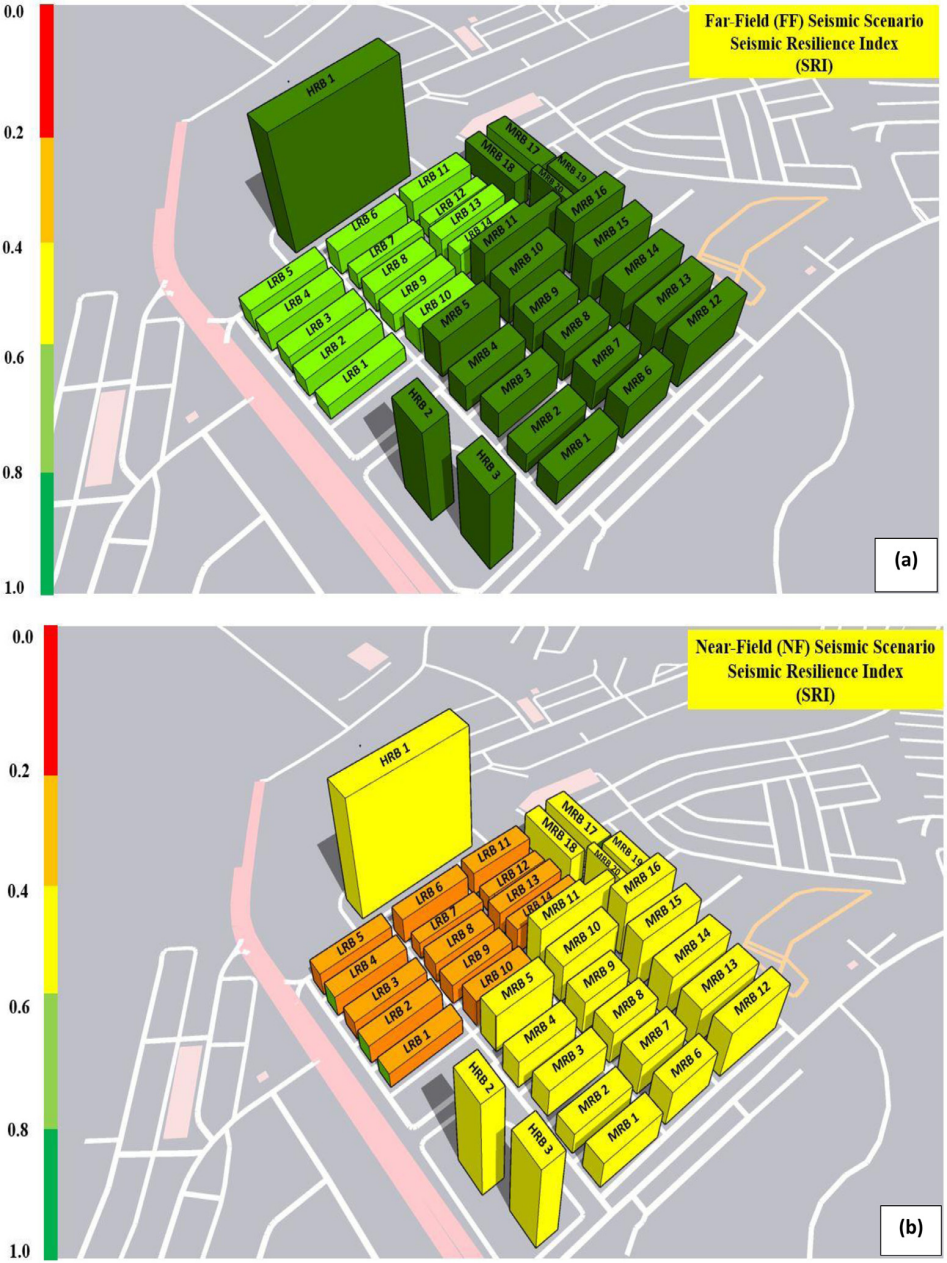
SRI values for the selected RC-buildings in case of (a): far-field, and (b) near-field seismic scenarios.

Additionally, Fig. 15 of the GIS mapping provides an illustration of the seismic resilience indices for the 25 structures that were selected. The SRI values for low-rise buildings range somewhere between 0.6 to 0.8, while those for mid- and high-rise buildings ranges between 0.8 and 1.0. This is applicable in the event of a far-field seismic scenario. In contrast, the SRI values for low-rise buildings are in the range of 0.2 to 0.4, while those for mid- and high-rise buildings are in the range of 0.6 to 0.8. This is the case for a scenario involving a near-field seismic event. It is clear that, in comparison to mid-rise and high-rise buildings, low-rise buildings have significantly less resistance and more susceptibility to damage.

## Conclusion

The concept of the seismic resilience is the key in the rescue and recovery processes for the community systems and structures after seismic excitation by evaluating the affected systems or structures’ post-seismic functionality.

Evaluation of seismic resiliency of the structures is an important step for government or relevant authorities of Malaysia or any other country to estimate or forecast the ability and functionality of the community systems or structures including both existing or under-construction structures that were designed in accordance of British Standard which have not taken the consideration of the seismic effects in the design process.

The assessment of the seismic resiliency of the systems and structures allows decision-makers to introduce the constructive recovery or rescue plans which have a balance efficacious in terms of repair time and repair cost. The main purpose of the research is to develop seismic resilience index (SRI) for the existing buildings in Malaysia after set of seismic events. Therefore, in this paper, a research methodology is proposed for assessing the seismic resilience of the existing reinforced concrete building in Malaysia using the seismic resilience index (SRI) approach which is modified from previous frameworks. The analysis is performed quantitatively and probabilistically for both near-fault and far-fault seismic scenario. Thus, the results are presented schematically in mapping form by using the Geographical Information System (GIS) platform.

According to the results of the two validation prototypes, in case of the far-fault seismic scenario, the buildings showed higher seismic resiliency and robustness compared to near-fault seismic scenario, which also indicated that the buildings experienced lesser direct losses and required shorter functionality recovery time. The major advantage of quantifying the seismic resiliency using probabilistic approach is to maximize the unexpected damages of the structures by generating the uncertainties caused by the variability in the mechanical properties of the structures. However, the challenging part of the proposed methodology is the analysis performed by the Finite Element (FE) platform which is time-consuming, and the information is required as detail as possible to obtain a higher accuracy outcome.

## Declaration of Competing Interests

The authors declare that they have no known competing financial interests or personal relationships that could have appeared to influence the work reported in this paper.

## Data Availability

No data was used for the research described in the article.
